# Supplementary figures and images for: Indole-3-acetic acid synthesized through the indole-3-pyruvate pathway promotes Candida tropicalis biofilm formation
Source: PLoS One. 2020 Dec 17;15(12):e0244246. doi: 10.1371/journal.pone.0244246 (PMC7746184; doi:10.1371/journal.pone.0244246)

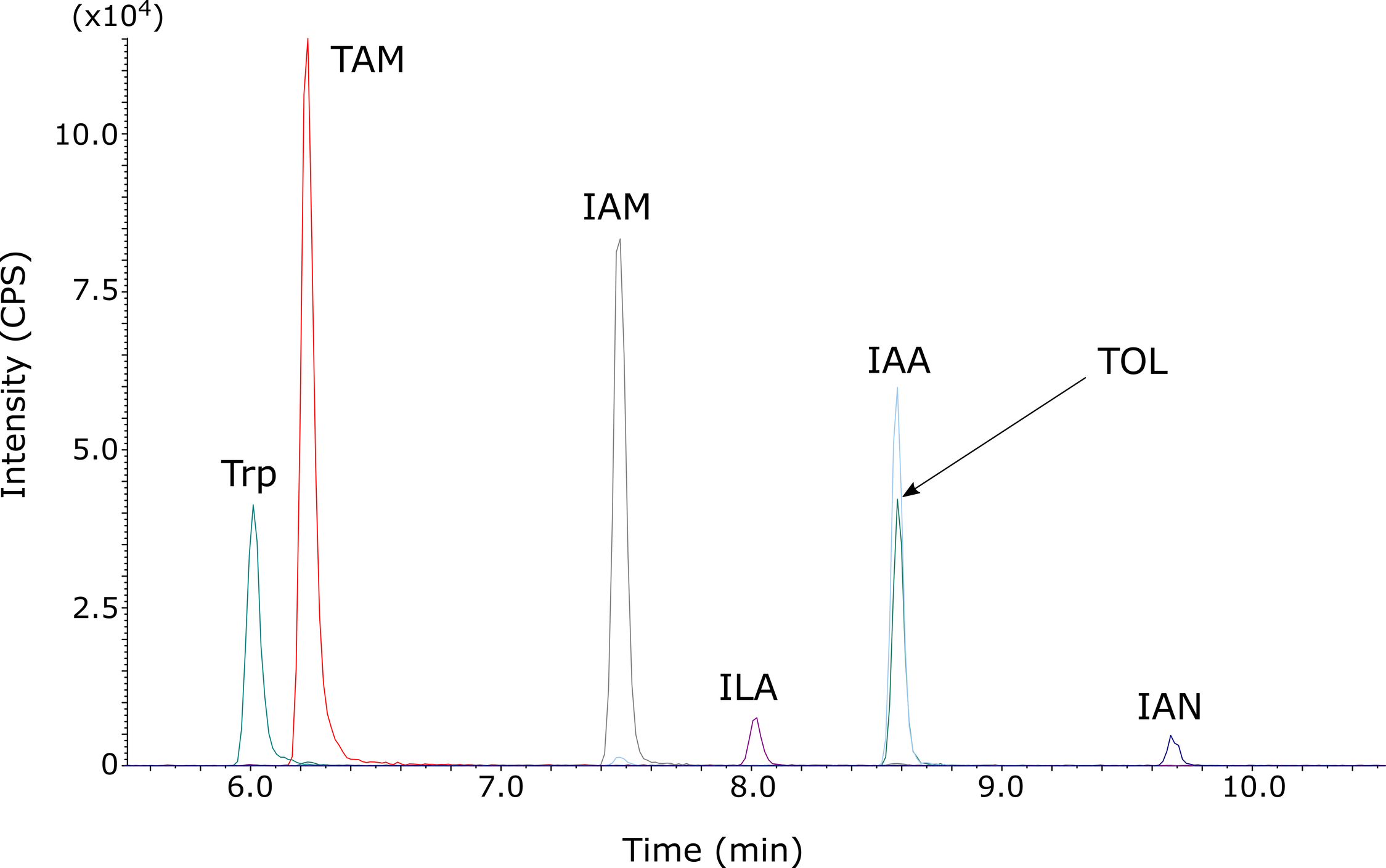

Supplement: S1 Fig — The concentrations of the metabolites are 1 nmol/mL. (TIF) [file pone.0244246.s001.tif]
